# Non-Human Primate Models of Enteric Viral Infections

**DOI:** 10.3390/v10100544

**Published:** 2018-10-05

**Authors:** Karol Sestak

**Affiliations:** 1 Division of Microbiology, Tulane National Primate Research Center, Covington, LA 70433, USA; ksestak.tulane@gmail.com; Tel.: +1-985-502-2289; 2 PreCliniTria, LLC, Mandeville, LA 70471, USA

**Keywords:** enteric virus, non-human primate, virome, macaque, dysbiosis, enteritis, diarrhea, animal model, rotavirus, calicivirus, gut microbiome

## Abstract

There is an important role non-human primates (NHP) play in biomedical research. Phylogenetic proximity of any of the NHP species to *Homo sapiens* assures that much better translatability of research outcomes from model studies involving human diseases can be achieved than from those generated with other pre-clinical systems. Our group and others used during past two decades NHPs in research directed towards viral and autoimmune disorders of the gastrointestinal tract. This review summarizes progress made in the area of enteric viral infections including its applicability to human disease.

## 1. Introduction

**Figure 1:**
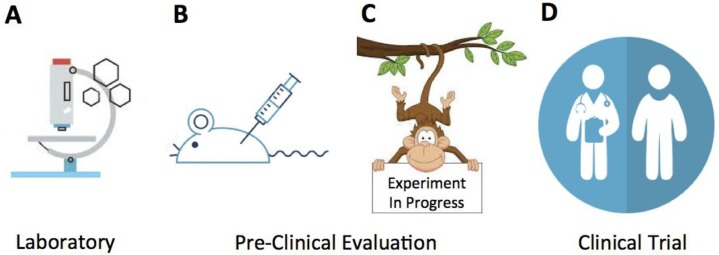
To maximize the potential of NHPs as pre-clinical models, it is essential to recognize function and information these models can generate in a cascade of events starting from: (**A**) the in vitro basic investigation; (**B**) concept validation; (**C**) pre-clinical trials; and ultimately (**D**) clinical studies.

The use of NHPs in biomedical research can be traced to early 20th century and discovery of ABO blood groups [1]. Since then, several of the NHP species, predominantly those kept in captivity, helped to facilitate progress in various biomedical areas including behavioral sciences, genetics/genomics, cancer, neuroscience, HIV/AIDS, cardiovascular and respiratory disorders, regenerative medicine, endocrinology, aging, immune-mediated disorders, and infectious diseases. From the infectious diseases point of view, simian model of AIDS was the one historically utilized most extensively and linked with seminal discoveries concerning retrovirus pathogenesis, viral transmission, reservoirs, vaccine and drug development [2] (Figure 1). In addition, there are emerging research areas exploiting those features of NHP models that cannot be easily duplicated in vitro or with alternative in vivo systems. In this short review, the human health-relevant enteric viruses of NHP host origin are discussed, as several of these represent human disease models of interest (https://nprc.org).

The main two groups of enteric viruses discussed and further specified in this section (simian rotaviruses and rhesus enteric caliciviruses) were derived from the following NHP species: Vervet Monkey (*Chlorocebus pygerythrus*), Rhesus Macaque (*Macaca mulatta*), and Pigtailed Macaque (*Macaca nemestrina*) [3,4,5,6,7,8,9,10,11]. Notwithstanding, the serological and direct virus detection evidence indicates that numerous other NHP species comprising those living in captivity and wilderness can be infected and/or seropositive [7,12].

## 2. Conventional and Specific Pathogen-Free (SPF) Research Colonies of Captive NHPs

To maintain the colonies of captive NHPs free of microbial pathogens, the following four “conventional” simian pathogens are being tested at the U.S. National Primate Research Centers to assure seronegative status: (1) Simian immunodeficiency virus (SIV); (2) Simian T lymphotropic virus type 1 (STLV); (3) Simian retrovirus type D (SRV); and (4) Herpes B virus. With continuous improvement of primate-specific diagnostic assays and increased demand for disease-free primates in biomedical research, some primate centers also raise expanded SPF colonies with voluntary diagnostic screening for following eight pathogens: (5) Simian foamy virus (SFV); (6) Primate cytomegalovirus (CMV); (7) Rhesus rhadinovirus (RRV); (8) Simian varicella virus (SVV); (9) Simian vacuolating virus 40 (SV40); (10) Lymphocytic choriomeningitis virus (LCV); (11) Measles virus; and (12) *Burkholderia psedomallei* (http://www2.tulane.edu/tnprc/microbiology/resources/).

## 3. Enteric Virus Infections in Captive NHPs

According to epidemiological surveys conducted during recent years by our group with participation of three National Primate Research Centers and three zoos in the U.S., a seasonal incidence of viral diarrhea-associated disease exists at captive NHP colonies [5,8]. Despite that viral diarrhea-linked morbidity is significant, current preventive measures do not target enteric viral pathogens as these persist in endemic form within the colonies as well as in the environment [5,13]. Understandably, the focus of the SPF eradication programs has been on blood-borne pathogens such as SIV, STLV, Herpes B, and others. Considering that annual diarrhea-associated losses of production were conservatively estimated by our group at hundreds of thousands USD per colony, it is evident that strategies that would help reduce such losses, and better define associated causes including viral diarrheas, would be of great scientific (translational) and economic (animal health and breeding) benefits.

## 4. The Common Enteric Virus Infections in Humans

Rotaviruses, along with noroviruses, are according to U.S. Centers for Disease Control and Prevention (CDC) the most common etiological agents of diarrhea in infants and young children worldwide. Both rotaviruses and noroviruses have also been isolated from monkeys, domestic mammals, plus rotaviruses also from birds and other species. It has been estimated that in Asia, Africa and Latin America more than 1 billion cases of diarrhea each year takes place with 2–3 million deaths. The majority of deaths occur in children less than five years of age. About half of all cases of severe diarrhea are caused by rotaviruses. As also reported by CDC, human noroviruses are annually worldwide responsible for more than one million hospitalizations and over 200,000 deaths in children less than five years of age. In the U.S. alone, an estimated 23 million cases of acute gastroenteritis, including 70,000 hospitalizations and 800 deaths, are attributed to human norovirus infections.

## 5. Simian Rotaviruses

The first rotavirus strain isolated from NHP species was the SA11 [3]. SA11 was derived from vervet monkey in South Africa in 1963 and used for decades in laboratories around the world as model to study the rotavirus replication and life cycle. Another simian rotavirus strain was derived from rhesus monkey in 1980 and named RRV [4]. The cell culture-adapted RRV was used for preparation of human-rhesus hybrid (reassortant) viruses that were employed by Wyeth Laboratories for formulation of first commercial rotavirus vaccine Rotashield in 1998. Despite its close to 100% efficacy, Rotashield had to be removed from the market in 1999 after it was determined that vaccination was associated with a 1:12,000 risk of intussusception in children. Subsequently, Rotashield was replaced by two attenuated, highly efficacious vaccines Rotarix by GlaxoSmithKline, and RotaTeq by Merck [14,15], which are still used today. Another two simian (NHP) rotavirus isolates, TUCH and PTRV, were derived from captive rhesus and pig-tailed macaques, respectively [5,6]. It was shown that all of the known simian rotaviruses thus far evolved by interspecies transmission and reassortment, i.e., by acquiring components of their RNA genome from the other, predominantly animal but also human rotaviruses [11]. Based on genotypic and phylogenetic comparisons between TUCH rotavirus structural proteins and cognate proteins of the representative rotavirus genotypes, the TUCH rotavirus belongs to a G3P[24] genotype while SA11 belongs to G3P[2], RRV to G3P[3], and PTRV to G8P[1] genotype [11].

When intragastrically inoculated into serum antibody-negative juvenile (<6 months-old) macaques, with no evidence of previous rotavirus infection, TUCH rotavirus was shown to consistently produce asymptomatic intestinal infection associated with peripheral, cell-mediated and antibody responses, as well as high levels of virus shedding [5,16]. The shedding in stools followed bell-shaped curve and decreased to baseline level within two weeks after experimental inoculation [5]. Symptoms of clinical diarrhea were inconsistent or absent. Hence, TUCH rotavirus infection model is referred to as “shedding” rather than “diarrhea” model [5]. Nevertheless, another study conducted in 1980 with a 141-day-old juvenile chimpanzee reported that when a SA11 simian rotavirus was orally administered to this animal, clinical symptoms of diarrhea and shedding took place over a period of nine days [17]. Based on these studies [5,16,17], it is thought that the pathogenesis of simian rotavirus infections is associated with small intestine although not causing severe enterocyte destruction and villous atrophy as seen in the case of rotavirus serum antibody-negative children.

RRV and TUCH rhesus-derived rotaviruses are currently being used in translational pathogenesis studies with murine model of biliary atresia, i.e., a neonatal obstructive cholangiopathy [18,19,20]. In pediatric patients, obstructive cholangiopathy often requires transplantation. Remarkably, the SRL peptide (TRTRVSRLY) motif on VP4 protein of RRV but not TUCH rotavirus was found to be responsible for binding to target cells in this model, i.e., murine cholangiocytes [20]. Further preclinical studies might need to be performed directly with NHP hosts to elucidate the exact nature of cellular and molecular interactions during rotavirus-induced biliary atresia. For example, it would be of interest to determine if RRV or TUCH infection of serum-negative newborn macaques produces obstructive cholangiopathy with symptoms of bilirubinuria, jaundice, acholic stools, and growth retardation, as seen in pediatric patients or BALB/c mice [20]. If such infection can be reproduced in NHPs, then synthetic, rotavirus VP4-binding, SRL-like peptides that were proven to ameliorate infection in murine model of biliary atresia, could also be evaluated for their potential as antivirals and immune response-modulators.

## 6. Rhesus Enteric Caliciviruses (ReCV)

No robust human-like models exist to study the pathogenesis and immunity of human noroviruses, although studies are being conducted with human volunteers, mice models and intestinal organoids [21,22,23,24,25,26,27,28,29]. A taxonomically newer group of enteric caliciviruses of rhesus monkey host origin with the name Recovirus (ReCV) was isolated and characterized by our group in 2008 [7,10]. ReCVs are closest relatives of human noroviruses and in contrast to noroviruses, can be grown in vitro. Epidemiological studies strongly indicate that ReCVs also infect humans [8,30]. Because of their biological properties, i.e., capability to grow in vitro and to cause diarrhea, ReCV macaque model is well positioned for studies where main objective is to elucidate specific aspects of enteric calicivirus pathogenesis and immunity.

According to conventional pathogenesis paradigm concerning enteric viruses, small intestinal epithelium is the primary target of infection. Nonetheless, most of the attempts to demonstrate replication of enteric caliciviruses inside the enteric epithelium failed. The confocal microscopy imaging of infected intestinal tissues revealed that mouse noroviruses are crossing the intestinal epithelium while being carried by some bacterial species of gut microflora that express virus-binding histo-blood group antigens (HBGA) [31]. It was proposed that HBGA-expressing bacteria carry noroviruses into deeper layers of intestinal wall where they enter the B and other cells [32]. Interestingly and coincidentally, few years prior to revelation of these important norovirus pathogenesis features, it was demonstrated that in human norovirus-challenged chimpanzees as well as in ReCV-challenged rhesus macaques, viral antigens appear not within but beneath the small intestinal epithelium—inside the lamina propria [33,34]. Despite progress made in recent years, it is still not clear what exact genera/species of intestinal bacteria are facilitating enteric calicivirus transcytosis and systemic spread. It is also not clear if B cells serve as primary targets of infection or only as virus carriers. Giving the low rate of virus replication in these cells, it is more likely that B cells are not the targets but only vehicles to carry the virus into other tissues. As ReCV macaque model would enable the time-scaled experiments during which collections of intestinal biopsies, stools, peripheral blood and urine samples can be carried out, ReCV model has strong potential to facilitate further discoveries.

As the example of such experiments, ReCV macaque model could be used to narrow down the taxa of primate gut microbiome that were suggested to facilitate transcytosis of human noroviruses across small intestinal epithelium [31]. We have evidence from the ReCV-unrelated studies that NHP gut microbiome can gradually change its diversity and composition with progression or remission of enteropathic disease [35]. Moreover, HBGA-defined macaques could be used to elucidate whether the particular host HBGA phenotype(s) predispose host to infection with particular ReCV type(s). ReCVs are genetically diverse group of viruses with multiple genotypes clustering within the four genetic types (GI.1, GI.2, GI.3, and GII.4) and two genogroups [9]. As demonstrated by recent studies [8,30], ReCVs can also infect humans. Furthermore, it was revealed with retesting of 500 rhesus stool samples collected originally in 2008 from Tulane National Primate Research Center that, beside ReCVs, these samples also contained GI, GII, and possibly GIV noroviruses, suggesting natural infections of captive NHPs with these clinically important viruses [36]. Thus, it might be possible to envision the emergence of cultivable recombinant human Noro-ReCVs that could potentially be exploited as vehicles for delivery of human norovirus vaccine antigens.

## 7. Advantages of Studies that Utilize NHP Models

Clinical studies with direct enrollment of patients are the important part of biomedical research. However, these studies need to be conducted in concert with pre-clinical trials (Figure 1) that utilize disease-prone animal models where samples including intestinal biopsies are obtained at desired and controlled experimental stages of the disease. The investigations with simian rotaviruses and ReCV-challenged macaques enable such experiments during which collections of peripheral blood, stools, saliva, urine samples, and intestinal biopsies are achieved with the help of specialized, NIH-funded Primate Research Facilities. Thus, we believe that NHP models possess, despite their relatively higher cost, significant advantages over other preclinical models. Besides obvious genetic and physiological significant differences with humans, more commonly used rodent models have an abbreviated and accelerated infancy; they develop relatively rapidly and become sexually mature within several weeks of age [37]. Infancy however is the critical time when many of the enteric viral (and other) diseases start to take hold in primates. Primate species develop slowly and do not reach puberty until the age of 3–4 (macaques) or 11–12 (humans) years [38]. Taken together, genetic and biological closeness of human and NHP species is the main reason we believe ethically conducted research with NHP models of human disease has an advantage over other in vitro- and in vivo-based models.

## 8. Other Enteric Viruses

From the taxonomic groups of enteric viruses with capability to cause gastroenteritis in humans and animals, the following were confirmed in biological specimens obtained from captive or semi-wild NHPs: enteric adenoviruses, enteroviruses, picobirnaviruses, coronaviruses, noroviruses, sapoviruses, astroviruses, anelloviruses, smacoviruses and parvoviruses [13,36,39,40,41,42]. It is important to emphasize that many of these viruses can be found not only in symptomatic (diarrheic) but mostly in asymptomatic animals. Thus, to suggest the pathogenic role in any species, experimental inoculations of virus-free subjects and reproduction of clinical or asymptomatic infection is required, consistent with previous studies [5,33,34].

## 9. Enteric Virome and Gut Dysbiosis

Several studies suggested that enteric virome might be in primates affected by immunodeficiency and/or autoimmunity in a putative gut dysbiosis fashion [43,44,45,46]. For example, in SIV-infected wild gorillas (*Gorilla gorilla*) and chimpanzees (*Pan troglodytes*), an abundance of viral families previously associated with gastrointestinal tract infections such as *Herpesviridae* and *Reoviridae* (gorillas), or *Circoviridae* and *Adenoviridae* (chimpanzees) were found increased [45,46]. This implies that selected enteric virome metrics might be utilized as markers of the disease progression/remission, and also be exploited for evaluation of novel therapies and vaccine approaches. Much remains to be discovered considering the complex nature of relationships concerning the factors influencing the gut microbiome composition, and the impact of gut dysbiosis on overall health.
